# Efficacy of Combined Highly Aspherical Lenslet Target (HALT) Spectacles and Low-Dose Atropine in Children with Poor Response to Atropine Monotherapy over 24 Months

**DOI:** 10.3390/jcm15208116

**Published:** 2026-10-19

**Authors:** Bryan Xiangrong Sim, Loh Kai-Lyn, Pauline Poh Lin Chan, Yudah Sri, Audrey Chia

**Affiliations:** 1 Singapore National Eye Centre (SNEC), Singapore 168751, Singapore; kailynloh@gmail.com (L.K.-L.); pauline.chan.p.l@snec.com.sg (P.P.L.C.); sri.yudah@snec.com.sg (Y.S.); audrey.chia.w.l@singhealth.com.sg (A.C.); 2 Singapore Eye Research Institute (SERI), Singapore 169856, Singapore

**Keywords:** combination, glasses, atropine, myopia

## Abstract

**Objectives**: To evaluate the 24-month efficacy of combining highly aspherical lenslet target (HALT) spectacle lenses with low-dose atropine (LDA) in children with poor response to atropine monotherapy. **Methods**: This prospective interventional cohort study included myopic children aged 6–11 years who continued to progress despite LDA (0.01–0.025%). Fifty children completed 12 months of follow-up and 48 completed 24 months. At 12 months, atropine dosage was maintained, reduced, or increased according to clinical discretion. Cycloplegic spherical equivalent refraction (SER) and axial length (AL) were measured at baseline, 12 months, and 24 months. **Results**: Myopia progression during the first year (SER: −0.07 ± 0.45 D; AL: 0.13 ± 0.14 mm) was markedly lower than during the 6 months before treatment (SER: −0.61 ± 0.39 D; AL: 0.24 ± 0.12 mm). During the second year, atropine dosage was reduced in 23%, maintained in 67%, and increased in 10% of participants. Mean second-year progression was −0.26 ± 0.41 D and 0.17 ± 0.17 mm, with no significant differences between treatment groups. Although second-year SER progression exceeded first-year progression (*p* = 0.003), both SER and AL progression remained significantly lower than pre-treatment rates (*p* = 0.004). Over 24 months, 73.9% of eyes progressed ≤ 0.50 D and 71.7% progressed ≤ 0.40 mm. Younger age and greater first-year progression independently predicted greater 24-month SER and AL progression. **Conclusions**: Myopia progression was relatively well sustained over 24 months after combination treatment was started in children previously progressing on LDA. Younger children and those with greater early progression may warrant closer monitoring.

## 1. Introduction

Myopia is a major and growing global public health problem, particularly in East and Southeast Asia, where prevalence in young adults exceeds 80% and 20–30% may develop high myopia [1,2,3,4]. Childhood myopia progression is primarily driven by excessive axial elongation and is associated with an increased lifetime risk of irreversible complications such as retinal detachment (RD), glaucoma, and myopic macular degeneration (MMD). Effective strategies to slow myopia progression during childhood are therefore essential to reduce long-term visual morbidity. Current myopia management includes pharmacological treatment with low-dose atropine (LDA) [5,6,7,8,9] and optical interventions designed to induce peripheral myopic defocus, such as highly aspherical lenslet target (HALT) [10,11] and defocus incorporated multiple segments (DIMS) [12,13] spectacle lenses. Both approaches have demonstrated substantial stand-alone efficacy in reducing spherical equivalent progression and axial elongation. However, treatment response is variable and a subset of children continues to progress despite LDA monotherapy, highlighting the need for alternative or adjunctive treatment strategies. Combination therapy targeting complementary mechanisms has emerged as a rational approach to enhance myopia control. In our previous 12-month study (Sim et al., 2025 [14]), adding HALT lenses to children inadequately controlled on LDA reduced myopia progression from a mean of −0.60 D or 0.24 mm to −0.15 D or 0.14 mm over 12 months, without significant adverse effects. The present study extends these observations to 24 months, evaluating the longer-term efficacy and safety of combination therapy and examining the effect of subsequent atropine dose adjustment, with a comparison to findings from randomized controlled trials and other combination studies.

## 2. Materials and Methods

A prospective interventional cohort study was conducted at the Singapore National Eye Centre. Patients were enrolled from March 2022 to July 2023. Inclusion criteria were children aged 6–11 years with ≥0.50 D progression over 6 months while on LDA (atropine 0.01% once nightly, atropine 0.01% twice daily or atropine 0.025% once nightly). Children had to be compliant with atropine treatment at recruitment and agreed to be compliant throughout the study period.

Exclusion criteria included ocular diseases other than myopia, strabismus, amblyopia, or prior contact lens therapy. Informed consent and assent were taken from the parent and patient respectively. The study was conducted in accordance with the tenets of the Declaration of Helsinki, and ethics approval was obtained from the SingHealth Institutional Review Board (IRB).

Participants continued their established LDA regimen and were prescribed Essilor Stellest (HALT) lenses. Cycloplegic refraction and axial length were measured at baseline and at 6, 12, and 24 months. Cycloplegic autorefraction was also measured at least 30 min after administration of 2 drops of cyclopentolate 1% (Alcon, Singapore) given 10 min apart at baseline, using a table-mounted autorefractor (CKR2, Topcon Corporation, Tokyo, Japan). Five readings, each less than 0.25 D apart, were averaged. AL was measured using the IOLMaster 500 (Carl Zeiss AG, Oberkochen, Germany). Five readings, each less than 0.10 mm apart, were averaged.

Treatment was maintained unchanged for the first 12 months but, subsequently, atropine dose/frequency could be modified based on clinical discretion (i.e., decreased, maintained, or increased). In general, atropine dose/frequency could be tapered/decreased when patients were older with stable myopia and increased when their myopia progressed. Primary outcomes were changes in spherical equivalent (SER) and axial length (AL) over 24 months.

Participants were divided into 3 groups—where atropine treatment was decreased (i.e., tapered or stopped, Group A), continued unchanged (Group B), or increased (Group C). Differences in baseline age, gender, race, baseline SER and AL and changes in SER and AL 6 months prior, in first year, second year and over 2 years were compared between groups. Paired and independent *t*-tests were used to compare changes between time periods.

Univariate analysis, with the change in SER and AL over 24 months and change between 12 and 24 months (dependent variables), was used to evaluate associations with age, sex, and SER and AL changes, using cluster analysis to account for inter-eye correlation,. Multivariable regression models were constructed to identify independent predictors after adjustments for age, gender, race, and treatment changes. Statistical significance was defined as *p* < 0.05. All analyses were conducted using STATA/IC version 11.2 (StataCorp, College Station, TX, USA) and IBM SPSS Statistics for Windows, version 29.0.2.0 (IBM Corp., Armonk, NY, USA).

## 3. Results

Fifty children were enrolled in the original study. Two children (four eyes) without the full 24-month follow-up were excluded from this study. In the remaining 48 children (96 eyes), baseline age was 9.0 ± 1.1 years, with more children being ethnic Chinese (85%) and female (65%). Across all eyes, first-year progression (−0.07 ± 0.45 D; 0.13 ± 0.14 mm) was much less than the prior 6 months of atropine alone (−0.61 ± 0.39 D; 0.24 ± 0.12 mm).

In the second year, 23% of eyes had atropine dose either tapered (n = 18 eyes) or stopped (n = 4 eyes) (group A), 67% maintained (group B), and 10% increased (group C). Children in Group A were significantly younger than those in group B (Table 1). Otherwise, baseline SER and AL were very similar between groups. Six children (12 eyes) switched from HALT lenses to alternative myopia-control lenses in the second year (two from Group A and four from group B). The reasons for this change included increased myopia (n = 3), cost (n = 2) and wear and tear of the lenses (n = 1).

**Figure 1:**
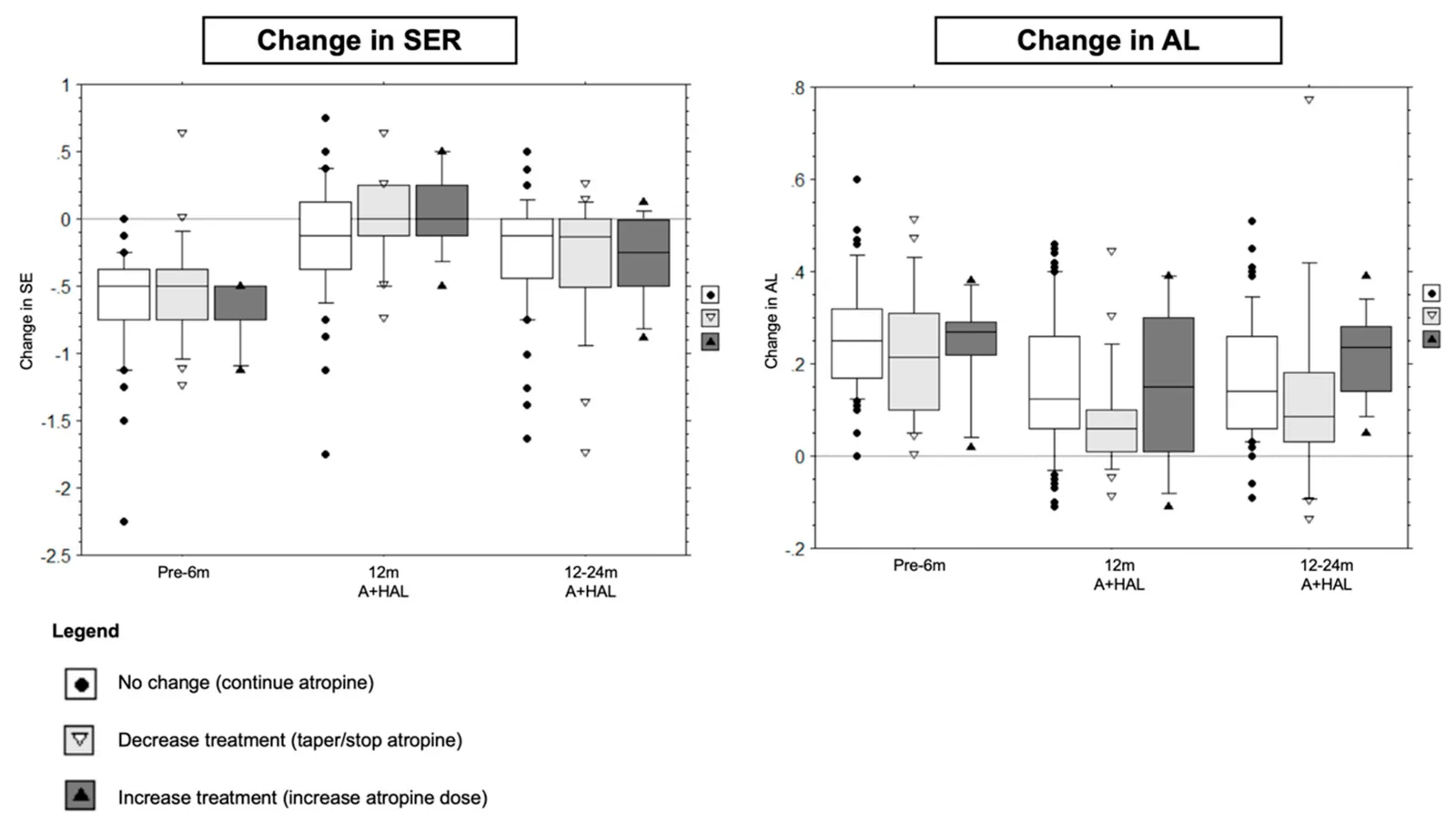
Box plot showing the distribution of spherical equivalent (SER) and axial length (AL) changes before and after HALT–atropine combination therapy according to atropine treatment adjustment strategy in the pre-6, 12 and 24 month periods. Similar findings were noted when only participants still wearing HALT lenses at 24 months (n = 42) were analyzed separately (Table A1).

There was no difference in SER and AL progression over the second year between groups, with SER changes of −0.31 ± 0.50 D, −0.24 ± 0.40 D and −0.30 ± 0.32 D (*p* = 0.495–0.937) and AL changes of 0.13 ± 0.25 mm, 0.17 ± 0.15 mm and 0.21 ± 0.10 mm (*p* = 0.256–0.541) in Groups A, B and C, respectively (Table 1). There was also no significant difference in the change in SER and AL between groups. Second-year progression (−0.26 ± 0.41 D; 0.17 ± 0.17 mm) was significantly greater than in the first-year for SER (*p* = 0.003) but not AL (*p* = 0.133) (Table 1). However, this progression was still much lower than 6 months prior to the start of combination treatment (Figure 1).

Overall, 71 eyes (73.9%) progressed by ≤0.5 D, while 14 eyes (14.5%) progressed by ≥1.0 D over 2 years. There were 66 eyes (71.7%) that progressed ≤0.4 mm, while 15 (16.3%) progressed by ≥0.6 mm over 2 years.

Univariate analysis suggested that the greater change in SE and AL over 24 months was associated with the greater change in SER and AL over the first year, with the change in AL also associated with younger age (Table A2). These changes were not associated with sex, race, baseline SE or AL, the change in SE or AL 6 months prior, or the treatment group (Table A2). The change in SER in the second year was mainly associated with the change in AL in the 6 m prior to combination and in the first year. The change in AL over the second year was associated with age and the changes in SER and AL over the first 12 months.

Multivariate models were created, taking care not to include the changes in SER and AL, which were correlated, into the same model. The change in SER and AL over 24 months (dependent variable) was analyzed with models including gender, age, treatment group and the change in AL over the first 12 months (Model 1) and the change in SER over the first 12 months (Model 2). The analysis suggested that the change in SER and AL over 24 months was associated with a greater change in both SER and AL over the first 12 months, with the change in AL over 24 months also being associated with younger age (Table 2).

The change in SER and AL in the second year was analyzed in models including gender, age, the treatment group and the change in AL over the first 12 months (Model 3) and SER over the first 12 months (Model 4). The greater change in SER in the second year was associated with the greater change in AL over the first year while the greater change in AL in the second year was both associated with the greater change in AL and SER over the first year, with age no longer being relevant (Table 2). There were no complaints of glare or near or peripheral blur in children after starting the combination treatment.

## 4. Discussion

In this prospective cohort, 50 children who were still progressing by ≥0.5 D on low-dose atropine (0.01% or 0.025%) were started on highly aspherical lenslet target (HALT) lenses. In a previous publication, we reported the 12-month follow-up findings which demonstrated that the addition of HALT lenses resulted in a reduced progression from −0.60 D/+0.24 mm in the preceding 6 months to only −0.15 D/+0.14 mm over 12 months. In the second year, clinicians were allowed to alter the treatment according to their clinical judgment. In this paper, we report the findings in the 48 children who were still under follow-up at 24 months. Treatment remained unchanged in 67%, reduced in 23% and increased in 10%. Compared to the first year of treatment, there was significantly more change in SER in the second year (−0.26 D vs. −0.07 D, *p* = 0.003) but no significant change in AL (0.17 mm vs. 0.13 mm, *p* = 0.133). However, the overall change over 24 months was still much less than that noted 6 months prior to when treatment was started. This suggested that despite alterations in the atropine dose, there was a sustained benefit of the combination treatment over 24 months.

In the second year, changes in the atropine dose were made according to clinical judgment, introducing confounding by indication. Given the small group sizes, the absence of statistically significant differences between groups should not be taken as evidence of similar treatment effects.

Children enrolled in this study represent a special clinical group who were still progressing despite treatment with low-dose atropine (0.01% or 0.025%). The mean progression over the preceding 6 months was comparable to, or even greater than, the placebo or single-vision lens groups in many myopia-control trials and substantially greater than that reported for children treated with similar doses in other studies (Table 3). These children may therefore be either rapid progressors in whom the atropine dose was insufficient or children who were destined to be non or poor responders to atropine therapy. As such, this cohort differs from those enrolled in most atropine or spectacle lens trials, where participants were treatment-naïve and may have consisted of many who would respond well to atropine therapy. Differences in age, baseline myopia, the presence of any previous myopia treatment and study design between studies may also make direct comparisons difficult.

Following the introduction of HALT lenses, first-year AL elongation (+0.13 mm) was lower than that reported in the randomized HALT trial by Bao et al. [10] and comparable to that reported by Sankaridurg et al. [11], while SER progression (−0.07 D) was smaller than both studies (Table 3).

When compared with studies combining atropine with HALT or DIMS lenses, first-year axial elongation in our cohort (0.13 mm) was within the range reported by most studies (0.05–0.16 mm) [23,24,25,26,28,29] and better than several others (0.21–0.28 mm) [22,27]. Refractive progression was also among the lowest reported, being most comparable to Guemes-Villahoz et al. (ASPECT) [22], Zhao et al. [23] and Lee et al. [24] studies (Table 3). These comparisons should be interpreted cautiously given the differences in age, ethnicity, baseline myopia, atropine concentration or formulation and study design. Overall, however, there is growing evidence that combined optical–pharmacology treatments may be more effective than single treatments. These findings support a practical treatment-escalation strategy, where an optical intervention can be added to atropine, or vice versa, should myopia control be deemed inadequate.

There are few studies reporting the 24-month outcomes of atropine–spectacle lens combination therapy (Table 3). In this study, although progression in the second year was more than in the first year, it was still much lower than that observed before the combination treatment. The change in treatment in some children may have altered the trajectory of myopia progression, but overall, progression over 24 months compared favorably with the published studies of atropine or HALT monotherapy. This suggests that treatment could be modified according to children’s response over the first year. Interestingly, multivariate analysis suggests that the greater progression of SER and AL over 24 months and in the second year were most strongly associated with the greater change in AL over the first 12 months and to lesser degrees with the change in SER over the first 12 months and younger age (Table 2). This was likely a reflection of how AL change was more rapid in younger children [5,6,15,30], and highlights the importance of monitoring both SER and AL.

### 4.1. Clinical Implications

This study addresses the clinical problem that arises when children continue to progress while on low doses of atropine. One common cause of a poor response is poor compliance. If compliance is good, however, options include increasing atropine dose or frequency, although this may result in more blur and glare. Alternatively, as noted in this study, it is also possible to add an optical treatment (such as the HALT lenses). Combination therapy may also be particularly useful in younger children and those with rapid myopia progression.

That said, 14% of children may still progress > 1.0 D and 20% may progress > 0.5 mm over 24 months. Children who are also already progressing more quickly on treatment are likely to be at greater risk. The options are then, besides altering atropine dose, to better manage environmental triggers (e.g., lack of outdoor time) or swap to an alternate optical treatment (spectacles or contact lenses) or new light-based therapies.

### 4.2. Strengths and Weaknesses

The strengths of this study include its prospective design, high retention rate, standardized measurements, and extended 24-month follow-up. Importantly, it provides real-world data on the effect of treatment modification, an area that has received little attention in the existing myopia-control literature.

Several limitations should be acknowledged. There was no control or monotherapy group for the comparison and the effect measured may be that of HALT alone, rather than an additive effect of combination. Over the second year, the treatment in some children was changed, introducing possible selection bias. Those children progressing faster in the first year may have been started on a higher dose of atropine, which would blunt the overall progression in the second year. Similarly, rebound when atropine was tapered or stopped may have had the opposite effect. Compliance with atropine and spectacle lens wear was also not objectively monitored and the levels of compliance cannot be confirmed with certainty. The inclusion of both eyes from each participant in the analysis may also introduce a confounding effect.

## 5. Conclusions

In this cohort of children who had previously progressed on LDA, myopia progression remained relatively limited over 24 months after combination treatment was introduced. Without a concurrent atropine-only group, the additional effect of HALT and the durability of any treatment effect cannot be determined. Longer follow-ups and larger randomized trials with monotherapy and combination arms are needed to identify which children may benefit from combination treatment, its optimal duration, and how treatment can be safely tapered or stopped.

## Figures and Tables

**Table 1:** Baseline characteristics and baseline and changes in spherical equivalent (SER) and axial length (AL) in the previous 6 months, 12 months and 24 months within 3 treatment groups.

		A: Decrease Treatment	B: Unchanged Treatment	C: Increase Treatment	*p*-Value		
	Total	Taper/Stop Atropine		Increase Atropine	AvsB	AvsC	BvsC
No	96 eyes	22 eyes	64 eyes	10 eyes			
Age (years)	9.00 (1.17)	9.45 (1.78)	8.84 (0.91)	9.00 (0.94)	0.036	0.308	0.694
Male (%)	34/96 (35%)	14/22 (64%)	16/64 (25%)	4/10 (40%)		0.004	
Chinese (%)	82/96 (85%)	20/22 (91%)	52/64 (81%)	10/10 (100%)		0.203	
SER (D) Baseline Change pre6 m Change 1st yr Change 2nd yr Change 2 yr *p*-value pre6 m vs. 2nd yr *p*-value 1st vs. 2nd yr	−4.30 (1.27) −0.61 (0.39) −0.07 (0.45) −0.26 (0.41) −0.34 (0.64) *p* < 0.001 *p* = 0.003	−4.17 (1.18) −0.52 (0.40) 0.00 (0.30) −0.31 (0.50) −0.32 (0.65) *p* = 0.092 *p* = 0.013	−4.32 (1.36) −0.63 (0.40) −0.12 (0.50) −0.24 (0.40) −0.36 (0.69) *p* < 0.001 *p* = 0.150	−4.49 (0.97) −0.72 (0.22) 0.06 (0.30) −0.30 (0.32) −0.24 (0.28) *p* = 0.123 *p* = 0.018	0.635 0.302 0.285 0.495 0.768	0.521 0.274 0.692 0.937 0.750	0.707 0.567 0.223 0.682 0.568
AL (mm) Baseline Change pre6 m Change 1st yr Change 2nd yr Change 2 yr *p*-value pre6 m vs. 2nd yr *p*-value 1st vs. 2nd yr	25.00 (0.76) 0.24 (0.12) 0.13 (0.14) 0.17 (0.17) 0.31 (0.27) *p* = 0.004 *p* = 0.133	24.94 (0.58) 0.21 (0.14) 0.08 (0.11) 0.13 (0.25) 0.22 (0.30) *p* = 0.165 *p* = 0.376	25.05 (0.86) 0.25 (0.11) 0.15 (0.14) 0.17 (0.15) 0.33 (0.27) *p* = 0.011 *p* = 0.333	24.83 (0.30) 0.24 (0.12) 0.15 (0.18) 0.21 (0.10) 0.36 (0.21) *p* = 0.908 *p* = 0.351	0.588 0.271 0.042 0.360 0.096	0.699 0.692 0.211 0.256 0.136	0.409 0.803 0.933 0.541 0.723

Continuous variables are expressed as mean (standard deviation) and nominal values as number (percentage). yr: year.

**Table 2:** Multivariate analysis of change in spherical equivalent (SER) and axial length (AL) over 24 months and between 12 and 24 months (dependent variables) with demographic and SER and AL measures over time. Cluster analysis is used to account for the use of 2 eyes.

	Change SER over 24 m			Change AL over 24 m		
Model 1	coefficient	95% CI	*p*	coefficient	95% CI	*p*
Female (ref male)	−0.16	−0.37, 0.04	0.118	0.00	−0.05, 0.06	0.855
Age at baseline	0.08	−0.06, 0.22	0.264	−0.04	−0.10, 0.01	0.169
Change AL first 12 m	−2.21	−3.31, −1.10	<0.001	1.47	1.15, 1.79	<0.001
Ref (Decrease) - No change - Increase	−0.22 0.08	−0.57, 0.12 −0.27, 0.43	0.198 0.642	−0.02 −0.01	−0.14, 0.18 −0.08, 0.17	0.768 0.481
Model 2						
Female (ref male)	−0.12	−0.29, 0.05	0.164	−0.00	−0.10, 0.10	0.973
Age at baseline	0.09	−0.01, 0.28	0.086	−0.07	−0.13, −0.01	0.016
Change SE first 12 m	1.09	0.92, 1.26	<0.001	−0.22	−0.42, −0.01	0.037
Ref (Decrease) - No change - Increase	−0.19 −0.10	−0.51, 0.13 −0.31, 0.09	0.250 0.292	−0.04 0.08	−0.22, 0.13 −0.06, 0.23	0.633 0.255
	Change SER 12–24 m			Change AL 12–24 m		
Model 3						
Female (ref male)	−0.12	−0.28, 0.03	0.121	0.00	−0.05, 0.06	0.855
Age at baseline	0.07	−0.04, 0.15	0.199	−0.04	−0.10, 0.01	0.169
Change AL first 12 m	−0.97	−1.64, −0.31	0.005	0.47	0.15, 0.79	0.004
Ref (Decrease) - No change - Increase	−2.23 −0.09	−0.54, 0.06 −0.33, 0.14	0.124 0.445	0.02 0.04	−0.14, 0.18 −0.08, 0.17	0.768 0.481
Model 4						
Female (ref male)	−0.12	−0.29, 0.05	0.165	0.00	−0.5, 0.06	0.946
Age at baseline	0.09	−0.01, 0.28	0.085	−0.05	−0.11, 0.00	0.083
Change SER first 12 m	0.09	−0.07, 0.26	0.271	−0.09	−0.18, −0.00	0.032
Ref (Decrease) - No change - Increase	−0.19 −0.10	−0.51, 0.13 −0.31, 0.09	0.249 0.296	0.00 0.06	−0.15, 0.16 −0.02, 0.15	0.953 0.158

**Table 3:** Comparison of efficacy (one-year) with other low-dose atropine (LDA) or myopia control spectacle lens monotherapy in East Asian populations and with other combination studies.

Study	Study Population	Intervention	Change 12 m SE/AL	Change 24 m SE/AL
This study	Prospective. N48. Children still progressing on LDA with 24 m follow-up. Mean age 9.0 YO, mean SER −4.3 D	LDA HALT + LDA	6 m prior: −0.61 D/0.24 mm −0.07 D/0.13 mm	−0.34 D/0.31 mm
Randomized Controlled Trials involving low-dose atropine (LDA) in East Asian populations				
Chia 2012/2016 Singapore [6,15]	ATOM2. RCT, N400, mean age 9.6 YO, mean SER −4.7 D	A0.5% A0.1% A0.01%	−0.17 D/0.11 mm −0.31 D/0.13 mm −0.43 D/0.24 mm	−0.30 D/0.27 mm −0.38 D/0.28 mm −0.49 D/0.41 mm
Yam 2019/20/22 Hong Kong [5,16]	LAMP. RCT, N438, mean age 8.4 YO, mean SER −3.8 D	A0.05% A0.025% A0.01% Placebo	−0.27 D/0.20 mm −0.46 D/0.29 mm −0.59 D/0.36 mm −0.81 D/0.41 mm	−0.55 D/0.39 mm −0.73 D/0.50 mm −1.12 D/0.59 mm −0.99 D/0.59 mm
Wei 2020/2023 China [17,18]	RCT (cross-over extension), N220, mean age 9.8 YO, mean SER −2.58 D	A0.01% Placebo	−0.49 D/0.32 mm −0.76 D/0.41 mm	−0.78 D/0.39 mm −0.56 D/0.34 mm
Hieda 2021/23 [19] Japan	ATOM-J. RCT, 7 sites, N171, mean age 8.9 YO, mean SER −2.9 D	A0.01% Placebo	−0.69 D/0.35 mm −0.77 D/0.39 mm	−1.26 D/0.63 mm −1.48 D/0.77 mm
Chia 2023 [20] Singapore	APPLE. RCT, 2 sites, N99, mean age 8.5 YO, mean SER −3.0 D	A0.01% Placebo	−0.39 D/0.23 mm −0.55 D/0.36 mm	
Ohno-Matsui 2025 [21] Japan	ORANGE. RCT, 34 sites, N299, mean age 9.4 YO, mean SER −2.73 D	A0.025% A0.01% Placebo	−0.44 D −0.66 D −0.95 D	−1.02 D/0.51 mm −1.31 D/0.64 mm −1.65 D/0.74 mm
Randomized Controlled Trials involving HALT lenses in East Asian populations				
Bao 2022 [10], China	RCT, N157. 8–13 YO, mean age 10.4 YO, mean SER −2.53 D	HALT SVL	−0.66 D/0.34 mm −1.07 D/0.57 mm	−0.66 D/0.34 mm −1.46 D/0.69 mm
Sankaridurg 2023 [11], Vietnam	RCT, N119. 7–13 YO, mean age 11 YO, mean SER −3.42 D	HALT SVL	6 m data: −0.20 D/0.05 mm −0.33 D/0.14 mm	
Studies with combination atropine and optical (HALT or DIMS) treatment				
Guemes-Villahoz 2025 [22] Spain	ASPECT. RCT, N102, mean age 9.7 YO, mean SER −3.16 D	DIMS + A0.025% SVL + A0.025%	−0.09 D/0.07 mm −0.19 D/0.18 mm	
Zhao 2024 [23] China	Retrospective, N105, mean age 9.4 YO, baseline SER −0.75 to −5.00 D	HALT + A0.01% HALT SVL	−0.13 D/0.09 mm −0.25 D/0.19 mm −0.63 D/0.34 mm	
Lee 2025 [24], Taiwan	Retrospective, N160, mean age 9.7 YO, mean SER −2.5 D	DIMS + A0.125% DIMS + A0.01% DIMS	−0.16 D/0.06 mm −0.17 D/0.06 mm −0.30 D/0.13 mm	
Tang 2025 [25] China	Retrospective, N175, mean age 9.6 YO, mean SER −5.13 D (moderate-high myopia)	HALT + A0.01% HALT SVL	−0.33 D/0.21 mm −0.43 D/0.21 mm −0.52 D/0.28 mm	
Huang 2022 [26], China	Retrospective, N107, mean age 9.06 YO, mean SER −2.59 D	DIMS + A0.01% DIMS SVL	−0.49 D/0.28 mm −0.79 D/0.41 mm −1.07 D/0.52 mm	
Nucci 2023 [27], Italy	Prospective, N146, mean age 11.7 YO, mean SER −1.5 to −2.0 D	DIMS + A0.01% SVL	−0.30 D/0.05 mm −0.71 D/0.17 mm	
Cao 2025 [28], China	Retrospective, N341, mean age 8.9 YO, mean SER −1.14 to −2.16 D across groups	DIMS + A0.01% OK + A0.01% SVL + A0.01%	−0.22 D/0.16 mm 0.21 mm −0.58 D/0.40 mm	
Song 2025 [29] China	Prospective, N161, mean age 9.8 YO, SER −0.75 to −6.00 D	DIMS + A0.01% OK	−0.25 D/0.14 mm 0.20 mm	

Abbreviations: YO: year old; SE: spherical equivalent; AL: axial length; RCT: randomized controlled trial. SVL: single-vision lens; HALT: highly aspherical lenslet target; DIMS: defocus incorporated multiple segments; LDA: low-dose atropine; A: atropine; A0.01%: atropine 0.01%; A0.025%: atropine 0.025%; A0.05%: atropine 0.05%; OK: orthokeratology lens.

## Data Availability

The data supporting the findings of this study are available from the corresponding author upon reasonable request, subject to institutional approval. The data are not publicly available to protect participant privacy and confidentiality.

## Abbreviations

The following abbreviations are used in this manuscript:

AL	Axial length
D	Diopter
DIMS	Defocus Incorporated Multiple Segments
HALT	Highly Aspherical Lenslet Target
LDA	Low-dose atropine
MMD	Myopic macular degeneration
OK	Orthokeratology
RCT	Randomized controlled trial
RD	Retinal detachment
SER	Spherical equivalent refraction
SVL	Single-vision lens

## Appendix A

**Table A1:** Baseline characteristics and baseline and changes in spherical equivalent (SER) and axial length (AL) in the previous 6 months, 12 months and 24 months within 3 treatment groups—including only participants/eyes still on HALT at 24 months, i.e., those who changed to other myopia control spectacle lenses (e.g., DIMS) are removed.

		A: Decrease Treatment	B: Unchanged Treatment	C: Increase Treatment	*p*-Value		
	Total	Taper/Stop Atropine		Increase Atropine	A vs. B	A vs. C	B vs. C
No	84 eyes	18 eyes	56 eyes	10 eyes			
Age (years)	9.02 (1.17)	9.33 (1.87)	8.92 (0.89)	9.00 (0.94)	0.217	0.817	0.605
Male (%)	26/84 (31%)	12/18 (64%)	10/56 (18%)	4/10 (40%)		0.004	
Chinese (%)	70/84 (83%)	16/18 (88%)	44/56 (78%)	10/10 (100%)		0.190	
SER (D) Baseline Change pre6 m Change 1st yr Change 2nd yr Change 2 yr *p*-value pre6 m vs. 2nd yr *p*-value 1st vs. 2nd yr	−4.38 (1.26) −0.62 (0.40) −0.04 (0.45) −0.26 (0.43) −0.30 (0.66) *p* < 0.001 *p* = 0.017	−4.27 (1.13) −0.54 (0.43) 0.07 (0.27) −0.25 (0.53) −0.23 (0.65) *p* = 0.062 *p* = 0.032	−4.40 (1.35) −0.64 (0.41) −0.08 (0.51) −0.25 (0.42) −0.34 (0.72) *p* < 0.001 *p* = 0.164	−4.49 (0.97) −0.72 (0.22) 0.06 (0.30) −0.30 (0.32) −0.24 (0.28) *p* = 0.123 *p* = 0.018	0.732 0.360 0.365 0.988 0.556	0.268 0.332 0.758 0.817 0.965	0.628 0.635 0.374 0.754 0.657
AL (mm) Baseline Change pre6 m Change 1st yr Change 2nd yr Change 2 yr *p*-value pre6 m vs. 2nd yr *p*-value 1st vs. 2nd yr	25.02 (0.80) 0.25 (0.12) 0.12 (0.13) 0.16 (0.18) 0.29 (0.27) *p* = 0.002 *p* = 0.022	24.95 (0.63) 0.23 (0.15) 0.05 (0.08) 0.13 (0.28) 0.18 (0.31) *p* = 0.154 *p* = 0.267	25.07 (0.91) 0.26 (0.11) 0.14 (0.13) 0.17 (0.16) 0.31 (0.26) *p* = 0.004 *p* = 0.076	24.83 (0.30) 0.24 (0.12) 0.15 (0.18) 0.21 (0.10) 0.36 (0.21) *p* = 0.908 *p* = 0.351	0.627 0.352 0.017 0.425 0.099	0.568 0.879 0.072 0.361 0.120	0.420 0.633 0.837 0.412 0.548

**Table A2:** Univariate analysis of change in spherical equivalent (SER) and axial length (AL) over 24 months and between 12 and 24 months (dependent variables) with demographic and SER and AL measures over time. Cluster analysis is used to account for the use of 2 eyes.

	Change SER 24 m			Change AL 24 m		
	Coefficient	95% CI	*p*	Coefficient	95% CI	*p*
Female (ref: Male)	−0.08	−0.42, 0.16	0.646	0.01	−0.17, 0.15	0.877
Chinese (ref: Non-Chinese)	0.21	−0.24, 0.67	0.351	−0.05	−0.22, 0.11	0.515
Age at baseline	0.08	−0.03, 0.20	0.153	−0.08	−0.13, −0.04	<0.001
Baseline SER	−0.08	−0.18, 0.02	0.117	−0.02	−0.06, 0.02	0.362
Change SER 6 m prior	−0.06	−0.19, 0.06	0.309	−0.09	−0.21, 0.02	0.121
Change SER first 12 m	1.10	0.93, 1.27	<0.001	−0.23	−0.43, −0.03	0.020
Baseline AL	0.13	−0.07, 0.34	0.190	0.00	−0.07, 0.07	0.985
Change AL 6 m prior	0.22	−0.05, 0.51	0.111	0.44	−0.09, 0.98	0.105
Change AL first 12 m	−2.22	−3.24, −1.19	<0.001	1.54	1.26, 1.82	<0.001
Ref (Decrease) - No change - Increase	−0.04 0.12	−0.38, 0.47 −0.13, 0.39	0.824 0.335	−0.11 0.03	−0.24, 0.02 −0.15, 0.21	0.096 0.723
	Change SER 12–24 m			Change in AL 12–24 m		
Female (ref male)	−0.02	−0.26, 0.21	0.819	−0.01	−0.13, 0.03	0.731
Chinese (ref non-Chinese)	−0.07	−0.35, 0.19	0.566	0.01	−0.08, 0.08	0.958
Age at baseline	0.07	−0.01, 0.16	0.105	−0.05	−0.10, −0.01	0.024
Baseline SER	−0.08	−0.20, 0.04	0.203	0.01	−0.02, 0.02	0.954
Change SER 6 m prior	−0.08	−0.32, 0.15	0.502	−0.05	−0.14, 0.04	0.295
Change SER first 12 m	−0.10	−0.06, 0.27	0.237	−0.10	−0.19, −0.01	0.021
Baseline AL	0.13	−0.07, 0.34	0.190	−0.02	−0.81, 0.02	0.355
Change AL 6 m prior	−1.98	−3.52, −0.44	0.013	0.41	−0.05, 0.89	0.082
Change AL first 12 m	−2.22	−3.24, −1.19	<0.001	0.54	0.26, 0.82	<0.001
Ref (Decrease) - No change - Increase	−0.07 −0.05	−0.39, 0.24 −0.26, 0.14	0.655 0.571	0.04 0.03	−0.20, 0.12 −0.02, 0.10	0.261 0.698
